# Offering on‐site mammography in workplaces improved screening rates: Cluster randomized controlled trial

**DOI:** 10.1002/1348-9585.12389

**Published:** 2023-02-23

**Authors:** Azusa Shima, Hideo Tanaka, Tomonori Okamura, Tomofumi Nishikawa, Ayumi Morino, Kayo Godai, Yukako Tatsumi, Mizuki Kawahara, Maiko Kiyohara, Yuichiro Kawatsu, Takashi Kimura, Naomi Miyamatsu

**Affiliations:** ^1^ Department of Clinical Nursing Shiga University of Medical Science Shiga Japan; ^2^ Occupational Health Care Office, Heiwado Co. Shiga Japan; ^3^ Public Health Center of Neyagawa City Osaka Japan; ^4^ Department of Preventive Medicine and Public Health Keio University School of Medicine Tokyo Japan; ^5^ Department of Health and Nutrition Kyoto Koka Women's University Kyoto Japan; ^6^ Department of Health Promotion Science Osaka University Graduate School of Medicine Osaka Japan; ^7^ Department of Hygiene and Public Health Teikyo University School of Medicine Tokyo Japan; ^8^ General Incorporated Foundation Kinki Health Administration Center Shiga Japan

**Keywords:** breast cancer screening rate, cluster randomized controlled trial, occupational health, on‐site mammography

## Abstract

**Objectives:**

Despite evidence of breast cancer screening efficacy, the screening rate has remained less than 50% in Japan. This study aimed to evaluate the effect of an environmental approach offering on‐site mammography in workplaces.

**Methods:**

Supermarket stores were randomly assigned into two groups, the intervention group (leaflet and mammography) and the control group (leaflet). From May to July 2018, participants in the intervention group were given a leaflet informing them of the subsidies for breast cancer screening and offered the opportunity to have mammography in their workplaces. Participants in the control group were given the same leaflet, but had to arrange their own screening outside the workplace. The primary outcome was the breast cancer screening rate in 2018. The odds ratio (OR) and 95% confidence interval (CI) for having screening in the intervention group compared with the control group were estimated using multilevel logistic regression.

**Results:**

We analyzed data from 1624 participants (mean age 53 years) from 25 supermarket stores (intervention: 8 stores, control: 17 stores). Among participants who had not attended screening in the previous year, the screening rate was 7% in the control group and 53% in the intervention group, with an adjusted OR (95% CI) of 14.22 (8.97–22.54). The effect was greater in those who had never attended screening before.

**Conclusion:**

In a worksite‐based cluster randomized controlled trial in Japanese supermarket stores, an environmental approach offering mammography in workplaces substantially increased the breast cancer screening rate within 1 year (UMIN000030465).

## 1 INTRODUCTION

Breast cancer is the most common malignancy among women worldwide.^1^, ^2^ In Asian countries, including Japan, the incidence rate of breast cancer has increased rapidly in the last few decades, with a sharp increase from the 1930s. Breast cancer is prevalent in the working‐age population.^3^ Early detection using mammography is very important because breast cancer causes various problems related to work and life, irrespective of survival.^4^, ^5^, ^6^, ^7^, ^8^ However, the Japanese breast cancer screening rate remained at 47.4% in 2019.^9^ This is lower than in many Western countries, despite various educational approaches by municipalities and policies reducing out‐of‐pocket costs.^10^, ^11^, ^12^, ^13^ In recent years, the focus has been on environmental approaches to increase health behaviors, including screening behaviors. These approaches attempt to reduce barriers to health behaviors by changing the environment rather than by educating people.^14^ In Japan, lack of time has been reported as a major barrier among female employees who have not had breast cancer screening.^15^, ^16^ On‐site mobile mammography can help reduce barriers for employed women.^17^, ^18^, ^19^, ^20^ However, breast cancer screening is generally provided by local governments in Japan, and we could not find any scientific reports about the effectiveness of on‐site mammography in workplaces. This study therefore aimed to evaluate the effect of an environmental approach offering on‐site mammography to Japanese female employees in a real‐world setting.

## 2 METHODS

### 2.1 Breast cancer screening system in Japan and the study setting

The Japanese guidelines for breast cancer screening recommend mammography every 2 years for women over 40 years old, because the highest incidence rate in Japan is in women aged 45–49 years.^7^, ^21^ Mammographic breast cancer screening has been conducted by all municipalities since 2004, following the “Basic Law for the Promotion of Measures to Cope with Cancer.”^10^, ^12^ Most municipalities cover most of the cost of mammography with public funds, and people can undergo screening for no copayment or a few thousand yen at most if they choose the date, time, and medical institution for screening and make an appointment.^22^ Some companies and health insurance societies also offer breast cancer screening, but the details of this provision are unknown.^12^

This study was conducted in 25 large supermarket stores owned by a single retail company in western Japan. Approximately 80% of the store employees are part‐time and 20% full‐time. Employment insurance, health insurance, and pension benefits were the same regardless of employment status. The retail company and its health insurance society had been encouraging breast cancer screening for employees by providing subsidies of up to 5000 yen (approximately $40) per year. This covered the cost of breast cancer screening in most of the municipalities and medical providers.

### 2.2 Study design and procedure

This was a worksite‐based, parallel‐group, open‐label cluster randomized trial. The 25 large supermarket stores were randomly assigned in a 1:2 ratio to two groups, the intervention group (mammography and leaflet) and the control group (leaflet).

Prior to the randomization, consent was obtained from the health insurance society, the managers of the 25 stores, and individual employees. Eligible participants were female employees who were at least 40 years old and worked in one of the 25 stores. Participants were informed about the study in January–March 2018 and received a pre‐survey about their history of breast cancer and breast cancer screening. Written informed consent for data collection related to breast cancer, breast cancer screening, and health checkup data was obtained from all participants. Participants were blinded to the purpose of the study (i.e., to investigate the effect of offering mammography on breast cancer screening rate), to reduce confounding between grouping and screening behaviors.

An independent statistician generated the randomized assignment sequence for the stores, which were stratified using the rate of employees who had attended breast cancer screening in the last 2 years. Ethical approval was obtained from the Ethics Committee of Shiga University of Medical Science (C2017‐201). The study protocol was registered in the University Hospital Medical Information Network Clinical Trials Registry (UMIN000030465).

### 2.3 Interventions

The intervention was conducted by the site manager based on the assignment. In May 2018, participants in the intervention group were given a leaflet. The leaflet contained the message “Let's attend breast cancer screening once every 2 years” with an illustration on the front page (Appendix S1). On the other side was an application form to obtain the subsidy for breast cancer screening at any medical provider or in any municipality. A mammography van was sent to workplaces for 1–2 days during May and June 2018, separately from the annual health checkup, allowing participants to attend breast cancer screening while at work if they desired. The participants were informed that this cancer screening was a research project. Participants under 50 years old had two‐view mammography, and participants who were 50 years old or over had one‐view mammography. The out‐of‐pocket cost of mammography was set at 1000 yen, which was almost the same as the cost in local governments. The 1000 yen could later be reclaimed as a subsidy. The reason for this procedure was to make the cost and process of reclaiming as similar as possible to receive a subsidized mammogram outside the workplace. Participants in the control group were given the same leaflet in May 2018 but were not given an opportunity to attend mammography at their workplace. If the participants attended breast cancer screening outside the workplace, they could receive a subsidy for the cost of the screening of up to 5000 yen, as usual.

### 2.4 Outcome measures and data collection

The primary outcome was the breast cancer screening rate in 2018. This was evaluated using a self‐administered questionnaire at the annual health checkup conducted in the workplace between January and March 2019. Participants also provided their history of breast cancer screening, history of breast cancer both personally and in close relatives, and pregnancy during the follow‐up period. Information about age, employment status, smoking status, body mass index, and score for the Japanese version of the Kessler Psychological Distress Scale (K6) were extracted from the health checkup data with the consent of the participants. The K6 is a self‐report questionnaire containing six questions. The optimal cutoff point was estimated to be 4/5 for mood and anxiety disorders in the Japanese general population.^23^, ^24^ Participants were excluded from the analysis if they met any of the following criteria: (i) pregnancy between 2017 and 2019; (ii) a history of breast cancer.

In the original study design, we had planned to follow the effects of the intervention for 2 years (up to 2019). However, following the positive response to the on‐site mammography in the intervention group in 2018, the employer's health insurance society decided to start providing mammography screening sequentially to all the stores in 2019. We therefore had to close the observations for this study at that point.

### 2.5 Statistical analysis

We anticipated that 25 clusters (8 supermarket stores in the intervention group and 17 in the control group) would have more than 80% power (two‐tailed *α* level of 0.05) to detect a proportional difference of 22% in the breast cancer screening rate in 2018 between the intervention group (60%) and the control group (38%; estimated by adding natural increase and increase by leaflet to the 2013 National Survey of Living Standards).^25^ This assumption was based on an average cluster size of 81 and intra‐cluster correlation of 0.1, using operational data from the company, and conservative estimates of a 30% participant drop‐out rate from decisions not to participate, job changes, or retirement.

To compare baseline characteristics between the two groups, we used chi‐squared tests for dichotomous and categorical data and unpaired *t*‐tests for continuous data. We assessed the odds ratio (OR) with 95% confidence interval (CI) for the primary outcome (breast cancer screening rate in 2018) in the intervention group compared with the control group using multilevel logistic regression considering clusters (i.e., supermarket stores and the number of participants from each store). In the adjusted model, we adjusted for age and smoking status, because both were significantly different between groups despite the randomization. Breast cancer screening is recommended every 2 years in Japan, and we therefore conducted the analysis by history of breast cancer screening in 2017. For participants who were not screened in 2017 and expected to be screened in 2018, we used stratified analyses to examine the difference in the effect of the intervention by participants' characteristics. The stratified characteristics were age (40s/50s/60s), employment status (full‐time/part‐time), history of breast cancer screening (at least once before/never), smoking status (current smoker or not), obesity (body mass index <25, ≥25), and suspected mood or anxiety disorder (K‐6 distress scale: 0–4, 5–24).^23^, ^24^ All analyses used Stata 16 (Stata Corp LLC). All p‐values were two‐tailed and *P* < .05 was considered statistically significant.

## 3 RESULTS

**FIGURE 1:**
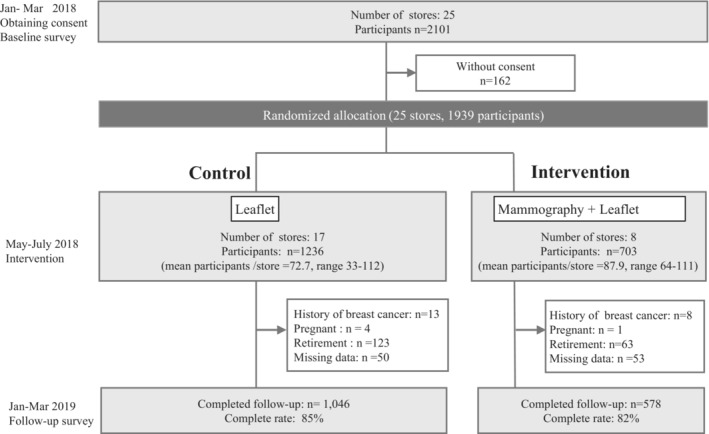
Flow of study. *Leaflet: a leaflet informing the subsidies for breast cancer screening. *Mammography: on‐site mammography was offered to the workplace on a voluntary basis.

Figure 1 shows the participant flow in this study. In total, 2101 eligible female employees were informed about the study and 1939 (92%) participants agreed to participate in January–March 2018. A total of 25 stores were randomized to the intervention group (8 stores) or the control group (17 stores). The interventions were conducted in May–July 2018. The follow‐up survey was conducted in January–March 2019 and 1624 employees (intervention group: *n* = 578, control group: *n* = 1046) completed the questionnaire. The average cluster size was 65 (range: 31–97).

Table 1 shows the characteristics of the participants in the two groups (with and without questionnaires completed from January to March 2019). The mean age (standard deviation) of the 1624 participants whose data were analyzed was 53.0 (7.1) years. Despite the randomized allocation, the mean age and prevalence of current smokers were significantly different between the two groups.

**TABLE 1:** Baseline characteristics of study participants, January–March 2018.

	Consented (*n* = 1913/1939)^a^			Completed questionnaire in 2019 (*n* = 1624)		
	Control	Intervention	*P*‐value	Control	Intervention	*P*‐value
Participants	1219	694		1046	578	
Age, years	53.6 (7.2)	53.1 (7.5)	.164	53.3 (7.0)	52.5 (7.3)	.048
Part‐time employment, *n* (%)	1075 (88.2)	629 (90.6)	.099	917 (87.7)	521 (90.1)	.134
BMI ≥ 25, *n* (%)	293 (24.0)	163 (23.5)	.786	251 (24.0)	133 (23.1)	.654
Current smoker, *n* (%)	195 (16.0)	143 (20.6)	.011	166 (15.9)	119 (20.6)	.017
History of breast cancer screening						
Screened in 2017, *n* (%)	163 (13.4)	106 (15.3)	.25	137 (13.1)	90 (15.6)	.169
Screened in 2016, *n* (%)	104 (8.5)	49 (7.1)	.254	90 (8.6)	40 (6.9)	.231
At least once before	578 (47.4)	301 (43.4)	.088	496 (47.4)	249 (43.1)	.093
K6 ≥ 5 point, *n* (%)	268 (22.0)	152 (21.9)	.966	231 (22.1)	128 (22.2)	.997
History of breast cancer in close relatives, *n* (%)	225 (18.5)	121 (17.4)	.842	210 (20.1)	108 (18.7)	.499

*Note*: Continuous data were analyzed using *t*‐tests and are shown as mean (standard deviation). Dichotomous data were analyzed using chi‐squared tests and are shown as number (percentage).

a We were unable to obtain health checkup data for 26 of the 1939 who consented, because they immediately moved to another company, so we analyzed data from 1913 people.

Table 2 shows the effect of offering mammography to workplaces on the breast cancer screening rate. Of the 1397 participants who did not have breast cancer screening in the previous year (2017) and were expected to be screened in 2018, 259 (53.1%) participants in the intervention group reported having breast cancer screening in 2018, as did 67 (7.4%) participants in the control group (adjusted OR: 14.22, 95% CI: 8.97–22.54).

**TABLE 2:** Effect of offering on‐site mammography on breast cancer screening rate in 2018 (*n* = 1624).

	Control	Intervention
Not having breast cancer screening in 2017, *n* = 1397		
Screening rate in 2018, *n* (%)	67/909 (7.4)	259/488 (53.1)
OR (95% CI)	1.00	13.90 (8.74–22.11)
Adjusted OR (95% CI)	1.00	14.22 (8.97–22.54)
Having breast cancer screening in 2017, *n* = 227		
Screening rate in 2018, *n* (%)	58/137 (42.3)	53/90 (58.9)
OR (95% CI)	1.00	2.00 (1.07–3.78)
Adjusted OR (95% CI)	1.00	2.03 (1.08–4.82)
Total, *n* = 1624		
Screening rate in 2018, *n* (%)	125/1046 (12.0)	312/578 (54.0)
OR (95% CI)	1.00	8.53 (5.62–12.96)
Adjusted OR (95% CI)	1.00	9.38 (6.07–14.50)

*Note*: Screening rate was assessed by a self‐administered questionnaire and analyzed using multilevel logistic regression considering clusters (i.e. stores and numbers of participants per store). OR (95% CI): Odds ratio and 95% confidence interval. Adjusted OR (95% CI): Odds ratio and 95% confidence interval adjusted for age, smoking status.

Table 3 shows the results of stratified analyses to investigate whether the effects of the intervention differed by the characteristics of the 1397 participants who did not have breast cancer screening in 2017. The adjusted ORs (95% CI) were 29.00 (15.24–55.18) in those who had never been screened and 10.84 (6.06–19.40) in those who had attended breast cancer screening at least once (*P* for interaction = .004). Age, employment status, smoking status, K‐6 distress scores, and obesity did not have any significant effects.

**TABLE 3:** Effects of intervention in participants who did not have breast cancer screening in 2017, by participants' characteristics (*n* = 1397).

	*n*	Breast cancer screening in 2018			
		Control	Intervention	OR (95% CI)	*P* for interaction
Ages					
40s	457	21/286 (7.3%)	99/171 (57.9%)	15.75 (9.08–27.33)	
50s	631	27/419 (6.4%)	117/212 (55.2%)	18.20 (8.75–37.87)	.745
60s	309	19/204 (9.3%)	43/105 (20.1%)	8.03 (3.67–17.59)	
Employment status					
Full‐time worker	155	11/110 (10.0%)	23/45 (51.1)	6.87 (2.80–16.89)	.276
Part‐time worker	1242	56/799 (7.0%)	236/443 (53.3)	15.71 (9.28–26.61)	
History of breast cancer screening					
At least once before	518	49/359 (13.7%)	101/159 (63.7%)	10.84 (6.06–19.40)	.004
Never	879	18/550 (3.3%)	158/329 (48.0%)	29.00 (15.24–55.18)	
Smoking status					
Non‐smoker	1136	61/758 (8.1%)	206/378 (54.5%)	13.41 (8.48–21.20)	.383
Current smoker	261	6/151 (4.0%)	53/110 (48.2%)	22.17 (7.98–61.58)	
K‐6 distress scale score					
0–4	1087	52/706 (7.4%)	204/381 (53.5%)	14.64 (8.87–24.18)	.822
5–24	310	15/203(7.4%)	55/107 (51.4%)	13.20 (6.54–26.65)	
Body mass index					
<25	1058	52/686 (7.6%)	189/372 (50.8%)	12.38 (7.50–20.46)	.882
≥25	339	15/223 (6.7%)	70/116 (60.3%)	21.86 (11.30–42.29)	

*Note*: Screening rates were assessed using self‐administered questionnaires. Odds ratios and 95% confidence intervals were assessed using multilevel logistic regression considering clusters (i.e., stores and numbers of participants per store) adjusting for age and smoking status.

## 4 DISCUSSION

In this study, the effects of an environmental approach offering on‐site mammography in workplaces were investigated in 25 supermarket stores in Japan, where the breast cancer screening rate in the previous 2 years was approximately 20% at baseline. On‐site mammography increased the breast cancer screening rate to 53% among participants who had not been screened in the previous year, with an absolute difference between the two groups of 46%. The results suggested that the intervention had a significant effect. In addition, the effect was greater in those who had never been screened and the effect was observed regardless of the characteristics of the participants.

Previous studies in workplaces in the United States have reported that on‐site mammography is an effective way to improve the screening rate.^17^, ^18^, ^19^ It has been recommended as a valuable strategy to reduce screening barriers for employees.^20^ One of the most important barriers to breast cancer screening that was removed in this study was time. In both the Japanese national survey and a previous observational study, the main reason given by women for not participating in cancer screening was a lack of time, mentioned by approximately 40% of the working‐age population. The frequency with which this reason was mentioned was notably higher than that of other reasons.^15^, ^16^ Those for whom time was the main barrier to cancer screening would have been screened if that issue had been removed.

Another barrier reduced in our study was inaccessibility. Previous studies have also reported that mobile mammography vans reduced the barrier of screening inaccessibility.^26^, ^27^, ^28^, ^29^ On‐site mammography in workplaces could have removed both the distance barrier and the psychological barrier. The option of on‐site mammography removes the problems of having to locate and travel to the screening site and take time off work. Some participants may also have been affected by the behavior of others, especially in a situation where approximately half their coworkers were having mammography. Our results are consistent with the findings of a cluster randomized trial in Chinese workplaces, in which breast cancer screening rates increased by approximately 60%. The Chinese study differed from ours in that it provided additional support to enable mammography outside the workplace instead of on‐site. In that study, the arrangement of appointments, transportation to the mammography site, workplace financial support, and release time for mammograms were managed to reduce barriers to screening.^30^ Our results therefore suggest that this environmental approach may be useful in reducing practical barriers to screening among employees.

In Japan, the breast cancer screening rate increased to 47% in 2019 through various efforts by municipalities, including traditional educational approaches, sending tailored messages, and removing out‐of‐pocket costs by the provision of free coupons.^10^, ^11^, ^13^, ^31^ As residents, the participants of our study would have benefited from some of these approaches. However, only approximately 20% had been screened in the last 2 years at baseline (the total number screened in 2016 and 2017; see Table 1). This was much lower than expected when the sample size was calculated. It is very interesting that on‐site mammography had a substantial effect in such a setting. In the stratified analysis, on‐site mammography was more effective in participants who had never been screened before. This was also consistent with another study that reported that on‐site mammography was particularly effective for some subgroups with low screening rates.^28^

Previous studies have reported that cancer screening rates are lower among populations such as smokers,^32^, ^33^, ^34^ part‐time workers,^35^ people with depression,^33^, ^36^ obese people,^37^ and those with low health literacy.^38^ There has also been concern that educational health promotion is less likely to be effective among individuals with low health interest and increased health disparities.^39^ However, our intervention was effective regardless of the characteristics of the participants. This suggests that this environmental approach using on‐site mammography is less likely to cause health disparities in the workplace.

The provision of mammography in workplaces incurs both the direct costs of screening and indirect costs, such as the human resources involved in arranging mammography and the time away from work to attend the examination (approximately 20 min, including walking to the mammography van, a brief medical interview, clothing removal, and the mammography examination). However, almost all the direct and indirect costs are for conducting the screening rather than educational costs to improve knowledge about screening and to promote health behaviors. Workplace screenings are probably more cost‐saving than employees booking a whole day off work to have a mammogram outside the workplace. At small sites, it may be difficult to recruit sufficient people to attend screenings and to find parking space for a mammography van, which takes approximately as much space as three cars. An environmental approach like that used in the present study may be feasible if several small sites in the neighborhood cooperate or if temporary police permission to use roads is obtained. We think it would be difficult to require all workplaces to cover the cost of screenings, especially small and medium‐sized enterprises and those with many part‐time employees. However, the employment rate of Japanese women in 2021 was approximately 70%,^40^ and the national breast cancer screening rate is therefore likely to improve if workplace mammography becomes more available. Financial support for employers and/or methods by which municipalities deliver screening to workplaces should be considered.

Our study had several limitations. First, the intervention was conducted from May to July 2018, and we asked about breast cancer screening in 2018 as the outcome. We may therefore have included screenings from January to March. However, we believe that this would have had a minimal effect on the results because the 2017 screening rates were similar for both groups, and the effect of the intervention was sufficiently significant. Second, the breast cancer screening rate was evaluated using a self‐administered questionnaire. This may have been subject to recall bias because the breast cancer screening rate in 2016 was smaller than in 2017. Participants' responses about screenings conducted more than 2 years ago may have been unreliable. To address this issue, we stratified results by past screening history, not limited to the previous 2 years. Third, we had to reduce the original 2‐year observation period to 1 year because the health insurance society independently decided to start breast cancer screening in all the supermarket stores once it became evident that so many of their employees wished to receive screenings in the workplace. We therefore focused our discussion on participants who had not been screened in the previous year. Although the study protocol had to be changed, the decision of the health insurance society to start its own initiative because of this trial was an important development. Fourth, despite randomization, there was an imbalance of participant characteristics between the assigned groups. To address the imbalances in age and smoking status, we used multivariable‐adjusted models for the main findings. Fifth, we did not evaluate the between‐group differences in cost‐effectiveness regarding future medical costs or changes in mortality, although the ultimate purpose of cancer screening is to reduce mortality. Sixth, the participants were limited to the employees of a single Japanese retail company. Further research in different fields is warranted to ensure generalizability.

Despite these limitations, this was, to the best of our knowledge, the first randomized trial in Japan showing the effect of an environmental approach offering on‐site mammography in the workplace. The results suggest that changing the healthcare system to provide breast cancer screening in the workplace would improve the early detection of breast cancer.

## AUTHOR CONTRIBUTIONS

Naomi Miyamatsu, Azusa Shima, Hideo Tanaka, Tomonori Okamura, Tomofumi Nishikawa, and Yukako Tatsumi conceived the ideas; Ayumi Morino and Yuichiro Kawatsu performed field site coordination; Kayo Godai and Takashi Kimura managed the intervention; Azusa Shima analyzed the data with support from Naomi Miyamatsu; Azusa Shima wrote the manuscript with support from Yukako Tatsumi, Mizuki Kawahara, and Maiko Kiyohara; All authors discussed the results and contributed to the final manuscript.

## FUNDING INFORMATION

This study was partly supported by a Grant‐in‐Aid for Scientific Research (B) (Grant Number 17K17539) from the Ministry of Education, Culture, Sports, Science and Technology of Japan.

## DISCLOSURE

*Approval of the Research Protocol*: Ethical approval was obtained from the Ethics Committee of Shiga University of Medical Science (C2017‐201). *Informed Consent*: Prior to the randomization, consent was obtained from the health insurance society, the managers of the 25 stores, and the individual employees. Written informed consent for data collection related to breast cancer, breast cancer screening, and health checkup data was obtained from all participants. Participants were blinded to the purpose of the study (i.e., to investigate the effect of offering mammography on breast cancer screening rate), to reduce confounding between grouping and screening behaviors. *Registry and the Registration No. of the Study/Trial*: The study protocol was registered in the University Hospital Medical Information Network Clinical Trials Registry (UMIN000030465). *Animal Studies*: N/A. *Conflict of Interest Statement*: Three authors (A.S., A.M., and Y.K.) are salaried employees of the retail company where this study was conducted. The other authors declare no conflicts of interest.

## Supporting information


Appendix S1.

## Data Availability

The data that support the findings of this study are available on request from the corresponding author. The data are not publicly available due to privacy or ethical restrictions.
