# Supplementary material for: Offering on‐site mammography in workplaces improved screening rates: Cluster randomized controlled trial
Source: J Occup Health. 2023 Feb 23;65(1):e12389. doi: 10.1002/1348-9585.12389 (PMC9950350; doi:10.1002/1348-9585.12389)
Supplement: Supplementary file 1 — Appendix S1. [file JOH2-65-e12389-s001.zip › JOH2_12389_6_Front page of the leaflet.pptx]

## Slide 1
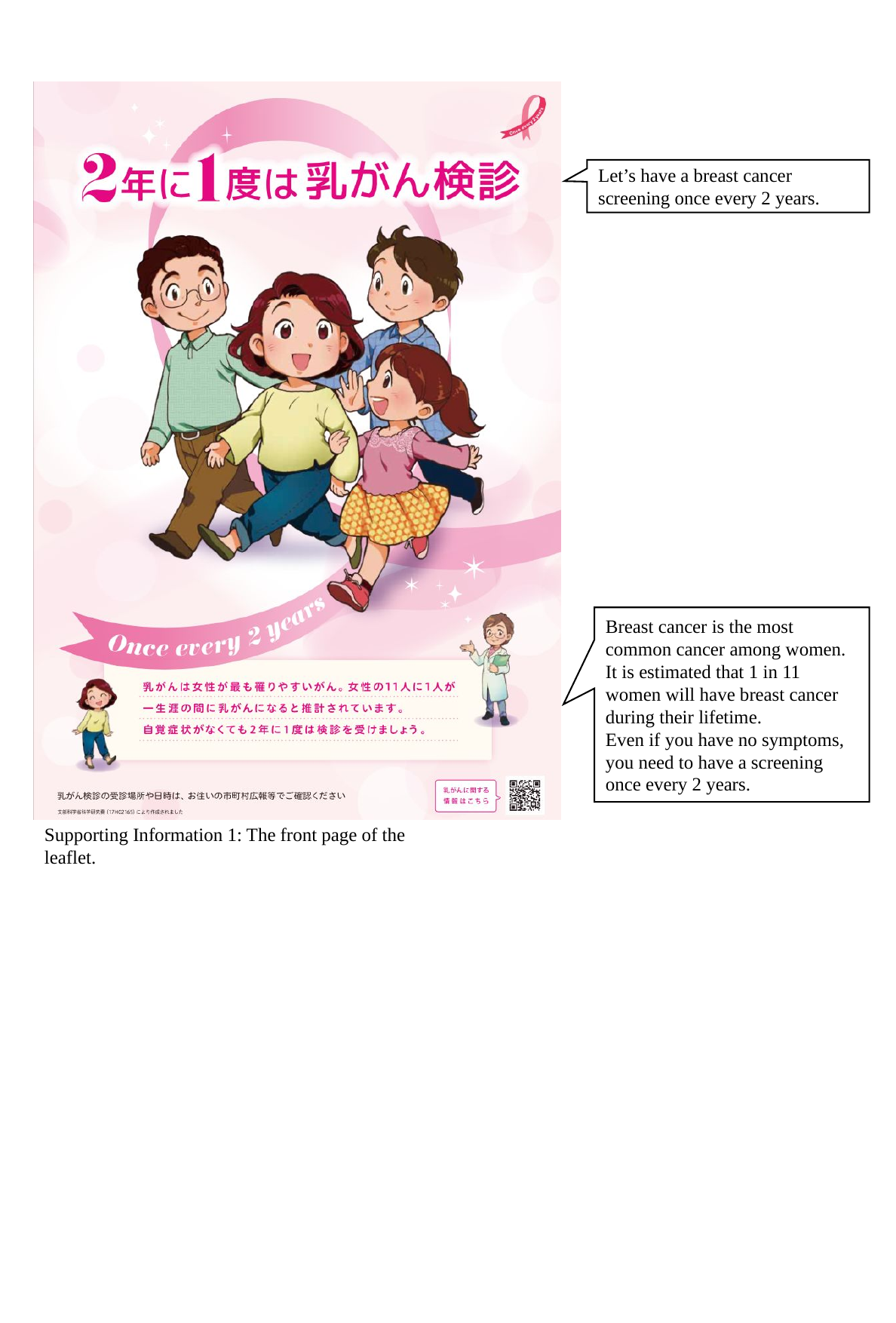

Let’s have a breast cancer screening once every 2 years.
Breast cancer is the most common cancer among women. It is estimated that 1 in 11 women will have breast cancer during their lifetime.
Even if you have no symptoms, you need to have a screening once every 2 years.
Supporting Information 1: The front page of the leaflet.
