# Supplementary material for: Offering on‐site mammography in workplaces improved screening rates: Cluster randomized controlled trial
Source: J Occup Health. 2023 Feb 23;65(1):e12389. doi: 10.1002/1348-9585.12389 (PMC9950350; doi:10.1002/1348-9585.12389)
Supplement: Supplementary file 1 — Appendix S1. [file JOH2-65-e12389-s001.zip › JOH2_12389_joh-2022-0352-oa-File006.pdf]

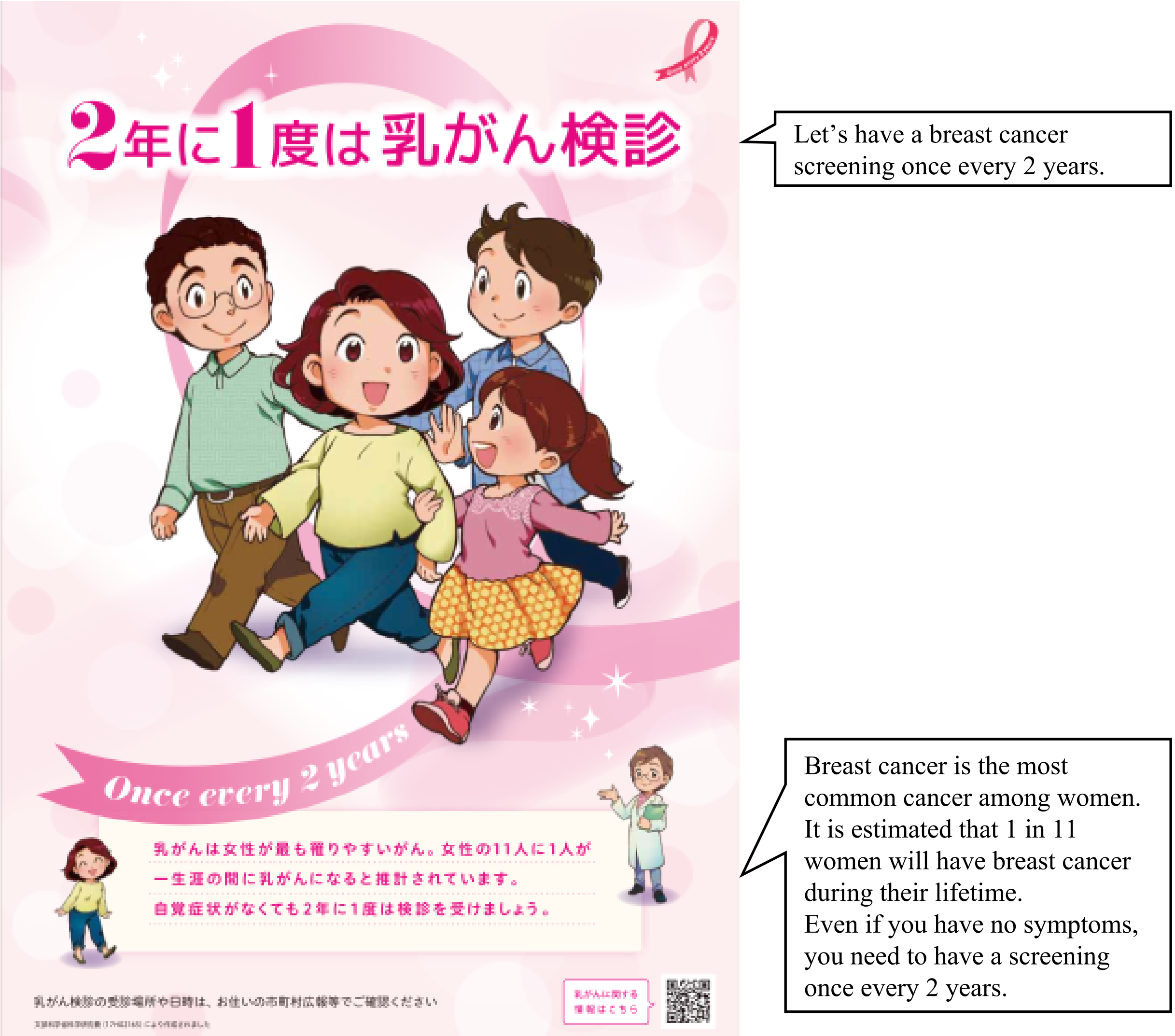

Supporting Information 1: The front page of the leaflet.
